# Restricted Access to Myeloid Cells Explained

**DOI:** 10.3390/v3091624

**Published:** 2011-09-05

**Authors:** Vicente Planelles

**Affiliations:** Division of Microbiology and Immunology, Department of Pathology, School of Medicine, University of Utah, Emma Eccles Jones Building, 15 North Medical Drive East 2100, Room 2520, Salt Lake City, UT 84112, USA; E-Mail: vicente.planelles@path.utah.edu; Tel.: +1-801-581-8655; Fax: +1-801-587-7799

**Keywords:** Vpx, DCAF-1, SAMHD1, ubiquitin, Cullin4, HIV, SIV, macrophage, dendritic cell, myeloid cell

## Abstract

The lentiviral accessory protein, Vpx, is known to counteract a restriction factor that is specific to myeloid cells, such as macrophages and dendritic cells. This review summarizes the findings in two seminal studies that identify SAMHD1 as the cellular protein that is responsible for myeloid cell restriction, and establish the existence of other types of restriction in these cells.

## 1. Introduction

The Genus “*Lentivirus*” includes viruses that infect mammals including primates, sheep, goats, cattle, horses and cats. Lentivirus infection has typically been associated with slow diseases affecting the immune system. Lentiviruses have complex genomes encoding multiple “accessory” genes in addition to the prototypical retroviral structural genes, *gag*, *pol* and *env*. Lentiviral accessory genes were initially found to be largely dispensable for virus replication *in vitro*. However, over the years, multiple biological roles have emerged for these genes, and their roles in *in vivo* viral replication and pathogenesis are increasingly becoming clear. Collectively, lentiviral accessory genes play multiple roles in regulating the relationship between the virus and the host and, more specifically, many of the encoded proteins specialize in overcoming innate immune defense mechanisms [1]. The host proteins that are effectors for these innate immune mechanisms are often referred to as “restriction factors”.

Lentiviruses are also well known for their unusual ability to infect non-dividing cells, such as myeloid-lineage cells. Thus, many lentiviruses have the ability to infect macrophages and/or dendritic cells. These cells are thought to play crucial roles in establishment of infection at mucosal sites, dissemination of virus throughout the body and, specifically lymphoid organs and the central nervous system, and to facilitate trans-infection of T-cells (reviewed in [2]).

**Figure 1.**
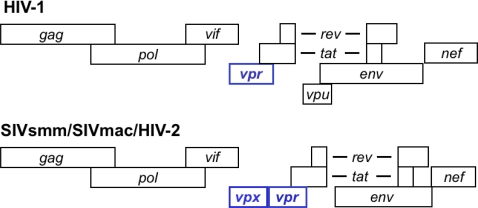
Genetic structure of HIV-1 and SIVsmm/SIVmac/HIV-2.

Primate lentiviruses naturally infect African primates. So far, 40 African primate species have been documented by serology to harbor lentiviruses, and 32 of those instances are confirmed by sequencing [3]. All primate lentiviruses encode an accessory gene termed *vpr* (viral protein, regulatory). Viruses in the phylogenetic group that includes SIVsm, HIV-2 and SIVmac contain two homologous genes, *vpr* and its paralog, *vpx* (Figure 1). It has been proposed that *vpr* and *vpx* in these viruses arose through duplication of an ancestral *vpr* gene [4]. This duplication would likely have occurred shortly after the HIV-1/SIVcpz and the SIVsmm/HIV-2 lineages diverged [4]. Because the SIVsmm/HIV-2 *vpx* genes appear to be more closely related to SIVagm *vpr* than they are to HIV-1 *vpr*, other investigators have proposed that *vpx* arose as the result of a recombination event that brought *vpr* sequences from SIVagm into SIVsmm, followed by a period of fast, adaptive evolution [5].

Vpr alleles from all tested lineages of primate lentiviruses share the ability to induce arrest in the G_2_ phase of the cell cycle [6–11], followed by apoptosis [12,13]. Vpx, however, has no effect on the cell cycle and, instead, is required for efficient infection of myeloid cells, such as macrophages and dendritic cells [8,14–16]. Vpx appears to promote the accumulation of full-length viral DNA in non-dividing cells [8,17–20]. To explain the ability of Vpx to enhance lentiviral infection of myeloid cells, Goujon *et al.* proposed that Vpx overcomes a restriction factor [17]. Treatment with proteasome inhibitors had a similar effect to that of Vpx expression, which led to the model that the restriction mechanism involved the ubiquitin/proteasome system [17].

Restriction factors are invariably genetically dominant. In agreement with that, fusion of permissive cells (*i.e.*, infection of which does not require Vpx) with restricting ones led to heterokaryons that had the restricting phenotype [19]. Another property of some restriction factors is that their activity is saturable (reviewed in [1]). Pre-delivery of Vpx protein into myeloid cells via SIV virions or virus-like particles (VLP) prior to infection dramatically enhanced the permissivity of myeloid cells to infection by either SIV or HIV-1 [19–22]. However, preincubation of target cells with either VLPs encoding no Vpx or encoding inactive mutants of Vpx did not render cells more susceptible to subsequent infection [22,23], even if VLPs were used at high multiplicities of infection [24]. Therefore, the available evidence indicates that the myeloid cell-specific restriction, presumably SAMHD1, is not saturable.

Despite the divergent functions of Vpr and Vpx, these proteins share their ability to bind and, presumably, manipulate the Cul4A^DDB1/DCAF1^ ubiquitin ligase (reviewed in [25]). Binding of lentiviral Vpr and Vpx proteins to DCAF1 is mediated by a highly conserved leucine-rich motif [26]. While the association with Cul4A^DDB1/DCAF1^ ubiquitin ligase was clearly required for Vpr to induce G_2_ arrest, the role of Vpx associating with the same ubiquitin ligase was, initially, unclear (reviewed in [25,27]). Two reports in 2008 demonstrated that the interaction of Vpx with DCAF1 is required for the enhancement of infectivity of myeloid cells [19,20]. These studies showed that depletion of DCAF1 via RNA interference overcame the restriction effect [19,20] and that expression of a Vpx mutant, Q76A, devoid of DCAF1 binding ability [20] was inactive.

HIV-1, a virus that does not encode Vpx, infects MDM (although with relatively low efficiency) and fails to infect DC. However, when pre-loaded with Vpx protein (via VLPs, for example) both MDM and DC become exquisitely sensitive to infection by HIV-1 [19–21]. Therefore, not only does Vpx overcome a restriction against cognate viruses (SIVsmm/SIVmac/HIV-2), but it also allows HIV-1 to infect DC, and to more efficiently infect MDM. Furthermore, inclusion of the Vpx protein HIV-1 particles enhanced infectivity of myeloid cells [28]. These observations demonstrated that a previously unrecognized restriction against HIV-1 exists in myeloid cells, although no activity in HIV-1 appears to have evolved against such a restriction.

Most cell lines fail to recapitulate the restriction that is observed in myeloid cells. One notable exception to that is the Thp-1 monocytoid line, which after differentiation in response to PMA stimulation, acquires macrophage-like properties. Differentiated Thp-1 cells restrict HIV-1 and SIVmac in a manner that is overcome by ectopic expression of Vpx [17,22].

## 2. Identification of SAMHD1 as the Vpx-Sensitive Restriction Factor

Recently, two groups have identified the culprit restriction factor that is overcome by Vpx as being the sterile alpha motif (SAM) and HD domain-containing protein-1 protein (SAMHD1) [29,30].

Laguette and coworkers established a stable transfectant of Thp-1 expressing SIVmac251 Vpx so that large numbers of cells expressing Vpx and the presumed restriction factor could be isolated. Cells were induced to differentiate, and extracts were prepared, and tandem-affinity purified using FLAG and HA tags on Vpx. Silver-stained bands on SDS-PAGE revealed the presence of multiple bands, which were identified via tandem mass spectrometry. Suspect bands included Vpx, components of the Cul4A^DDB1/DCAF1^ ubiquitin ligase complex and SAMHD1 [30].

The isolation of SAMHD1 by Hrecka and co-workers [29] followed a slightly different method. Hrecka *et al.* took advantage of the known requirement of the Cul4 complex for the function of Vpx, and transfected the major subunits of that complex (Cul4, DDB1 and DCAF1), along with SIVmac239 Vpx, into 293T cells. This is particularly remarkable given that SAMHD1-mediated restriction does not work in this cell line. Epitope tags for tandem affinity purification were place on distal members of the complex, specifically HA-Cul4 and FLAG-Vpx so that (a) partial complexes would not be purified; and (b) complexes containing DCAF subunits other than DCAF1 would also not be purified.

**Figure 2.**
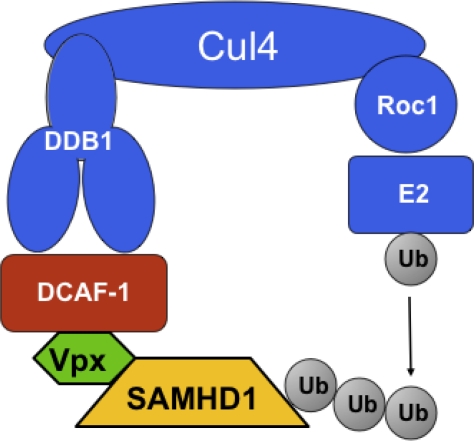
Proposed model for the ubiquitin ligase complex responsible for putative ubiquitination of SAMHD1. Adapted from [25].

Both research teams, using different methods as well as different cell types, produced the same hit, SAMHD1, as the candidate protein for the long-sought myeloid restriction factor. The expected properties of SAMHD1 in the context of Vpx restriction were confirmed as follows. First, both groups showed that Vpx induced proteolytic degradation of SAMHD1, which was overcome by incubation by proteasome inhibitors. Regarding the specific role that the Cul4/DDB1/DCAF1 complex is thought to have in degradation, both groups tested the role of Q76 Vpx residue, mutation of which was earlier shown to be unable to bind to DCAF1 [26] and not to be able to enforce restriction [20]. As expected, Vpx Q76A mutants in either SIVmac251 [30] or SIVmac239 [29] failed to induce degradation of SAMHD1. Hrecka *et al.* more specifically probed the role of Cul4A^DDB1/DCAF1^ by performing RNAi experiments in MDM, targeting DCAF1. These experiments showed that depletion of DCAF1 in MDM abolished Vpx’s ability to induce degradation of SAMHD1 [29]. A model describing the manipulation of Cul4A^DDB1/DCAF1^ by Vpx is presented in Figure 2.

SAMHD1 expression correlates with the ability or inability of various cell types to restrict, with some exceptions (see below). Thus, SAMHD1 is highly expressed in restricting cell types, such as Thp-1, monocytes, monocyte-derived macrophages (MDM) and monocyte-derived dendritic cells (MDDC) and is undetectable (by Western blot) in permissive ones, such as Jurkat, SupT1, HPV-ALL and U937 [30]. However, as pointed out by Hrecka and co-workers, this correlation is far from perfect [29]. This is exemplified by the observation that certain cell types, such as undifferentiated Thp-1 and 293T, which are unable to restrict, do express SAMHD1 [29]. Therefore, the presence or absence of SAMHD1 alone fails to completely explain the restriction of HIV-1 and SIV in myeloid cells, and therefore one anticipates that the story will get even more interesting as additional requirements, perhaps cellular co-factors, come to light.

The Vpr and Vpx family of accessory proteins is conserved in sequence but functionally diverse. To confirm whether the newly discovered ability of Vpx to induce degradation of SAMHD1 correlated with the known functional abilities of various alleles to overcome restriction in myeloid cells, both Laguette *et al.* and Hrecka *et al.* tested a variety of such alleles [29,30]. In summary, *vpx* alleles from SIVmac251, HIV-2_ROD_, and SIVmac239 induced degradation of SAMHD1 and overcame myeloid restriction to HIV-1 [29,30]. In contrast, *vpx* from two different isolates of SIVrcm from Gabon and Nigeria, or from HIV-1 *vpr* failed to instigate degradation of SAMHD1 [29,30].

Because Vpx induces degradation of SAMHD1, depletion via RNAi should phenocopy the expression of Vpx, and consequently render cells more permissive to infection. Laguette and colleagues showed that siRNAs directed against SAMHD1 indeed were able to render dendritic cells between 6- and 34-fold more sensitive to infection by various HIV-1-derived lentiviral vectors. Furthermore, overexpression of an siRNA-resistant cDNA of SAMHD1 restored the restriction phenotype [30]. Hrecka *et al.* found that siRNAs against SAMHD1 had a modest depletion effect, possibly indicating that SAMHD1 protein has a long half-life, and resorted to a two-step strategy for depletion of SAMHD1. In the first step, pre-existing SAMHD1 protein would be degraded via VLP delivery of Vpx. Four days later, cells were transfected with SAMHD1 specific siRNAs, in order to prevent *de novo* synthesis. This combined approach achieved the desired effect, and rendered macrophages sensitive to infection by HIV-1, SIVmac and HIV-2 [29].

As mentioned above, previous work had shown that Vpx-defective SIVmac and HIV-2 viruses, or HIV-1, when infecting dendritic cells, exhibited a defect in synthesis of late reverse transcripts, while a similar defect was not present in the synthesis of early reverse transcripts. Therefore, the expectation was that depletion of SAMHD1 would restore efficient synthesis of late reverse transcripts. Laguette *et al.* found that infection with an HIV-1-luciferase virus led to 13-fold higher levels of viral DNA accumulation in SAMHD1-silenced THP-1 cells when compared to control (scrambled siRNA) treated cells [30]. Hrecka *et al.* performed a more detailed reverse transcription analysis and demonstrated that, as expected, the presence of SAMHD1 in retroviral infection correlated with a selective decrease in the levels of late reverse transcripts but not of early ones. Furthermore, this effect was progressively attenuated with increasingly more effective SAMHD1-depleting treatments (a combination of VLP-Vpx delivery and siRNA) [29].

## 3. SAMHD1 and the Aicardi-Goutières Syndrome

The human SAMHD1 open reading frame was first identified in a cDNA library prepared in MDDC, as being the homolog of the mouse gene, MG11, which is inducible by IFN-γ in MDM [31,32]. The new gene was then termed DCIP for dendritic cell-derived interferon-γ induced protein [31].

SAMHD1’s HD domain is predicted to coordinate divalent-cations via two His (H) and two Asp (D) residues with variable spacing (H…DH…D) [33]. The HD domain is typically present in phosphohydrolases that can carry out phosphomonoesterase and phosphodiesterase activities [34]. The substrates for all studied HD domain proteins, with one exception, are nucleotides [33]. The *E. coli* YfbR protein for example, is an HD domain-containing enzyme that converts dNMPs to nucleosides and inorganic phosphate, thus controlling intracellular levels of nucleotides [33]. The exception is the YhaM protein from *Bacillus subtilis*, which has exoribonuclease activity [35]. Although the enzymatic activity of the SAMHD1 HD domain is not know at this time, when Laguette *et al.* mutated the canonical residues H and D to A and A, respectively, the resultant SAMHD1(HD/AA) was unable to induce restriction toward HIV-1 [30]. Therefore, the putative phosphohydrolase or exonuclease activity of the HD domain appears to be required for restriction.

The sterile alpha motif (SAM) is a 65–70 amino acid residue domain that has been implicated in protein-protein interactions [36] and, in one specific instance, in binding to RNA hairpins [37]. Rice *et al.* investigated the possibility that the SAM domain of SAMHD1 could interact with RNA hairpins by surface plasmon resonance and by *in-silico* modeling based on the available structure SAMHD1’s SAM domain, and obtained negative results [38]. This would suggest that SAMHD1’s SAM mediates interaction with another protein.

The gene encoding SAMHD1 is best known in the context of Aicardi-Goutières syndrome (AGS), a rare genetic disorder that causes encephalopathy and other sequelae that have striking resemblance to congenital infection and to some aspects of systemic lupus erythematosus (reviewed in [39]). The effector mechanism in the above diseases appears to be a type-I IFN-mediated innate immune response triggered by the presence of viral or host nucleic acids. AGS is genetically heterogeneous, with five loci (*AGS1-5)* so far having been identified as responsible for this syndrome when mutated. *AGS1-4* were identified as the (TREX1), RNASEH2B, RNASEH2C, RNASEH2A, respectively [39]. In 2009, *AGS5* was identified as the gene encoding SAMHD1 [38].

How the genes involved in AGS participate in its pathology is not well known. However, because TREX1 and RNASEH2 possess nucleolytic activity, it has been proposed that they participate in removing nucleic acids that may represent waste or the product of reverse transcription of transposable elements [40,41]. When these unwanted or aberrant nucleic acids are not cleared, they are thought to trigger a cell intrinsic type I IFN response. It is interesting to note that TREX1 has previously been implicated in the HIV-1 life cycle [42], although in a somewhat different manner to what Stetson *et al.* observed in the context of transposable elements [41]. Yan *et al.* proposed that TREX1 suppressed a type I IFN response that otherwise would be induced by HIV-1 infection [42]. TREX1 was proposed to degrade cytosolic DNA resulting from HIV-1 reverse transcription, which would otherwise be capable of inducing an IFN response [42].

## 4. Yet Another Restriction

Manel and co-workers investigated the fate of viral infection in the presence or absence of the myeloid cell restriction and, specifically, whether any antiviral signaling was triggered when restriction was surpassed [21]. When restriction was overcome via VLP pre-delivery of Vpx, HIV-1 infection triggered a potent type I IFN response that was dependent on *de novo* viral gene expression. Pertel and colleagues in a separate study also analyzed the properties of the IFN-induced inhibition of viral infection in MDDC, to find that VLP-delivered Vpx could overcome a potent block to HIV-1 infection, which was derived from type I IFN stimulation [43]. This block resulted, presumably, from activation of the same “cryptic sensor” reported by Manel *et al.* [21], and it did not appear to have the properties of the block imposed by SAMHD1. Instead, this type-I IFN-derived restriction became active only if infection was successful beyond SAMHD1’s restricting ability. There were several surprises in the Pertel study. First, relief of the HIV-1 block by Vpx was DCAF1-independent [43]. Thus, Vpx mutants devoid of DCAF1 binding (such as Q76A and R80F) exerted similar relief of restriction as wild-type Vpx. Also, Vpx effectively relieved restriction in the face of RNAi-mediated depletion of DCAF1 [43]. The second surprise in the studies by Pertel *et al.* was that Vpx, while able to render MDDC cells permissive to HIV-1 infection, failed to relieve the potent block on SIVmac and HIV-2 induced by IFN stimulation [43]. Triggering of this response required viral gene expression, specifically, Gag protein [43]. Therefore, two new levels of restriction are emerging in myeloid cells, which act sequentially. The first level of restriction limits infection at an early step of the viral life cycle (reverse transcription) and is effected by SAMHD1. Vpx can overcome SAMHD1 restriction via recruitment of Cul4A^DDB1/DCAF1^. A second level of restriction is triggered at a late stage in the viral life cycle and by an unknown sensor, and results in a potent type-I IFN response that very much resembles the response triggered by certain toll-like receptors. Vpx can help HIV-1 overcome this late response, but, paradoxically, is unable to facilitate infection by the cognate viruses, SIVmac and HIV-2.

Finally, it is worth mentioning here that APOBEC3A has been found to inhibit infection of myeloid cells by alpharetroviruses [44], and by lentiviruses, such as HIV-1 and SIVmac/HIV-2 [45,46]; and that Vpx compromised the stability of APOBEC3A [45,46]. The antiviral activity of APOBEC3A, however, appears to reveal a different type of restriction from that imposed by SAMHD1. Key differences are that APOBEC3A expression is upregulated by HIV-1 infection although in a type I IFN-independent fashion [45]; and that Vpx-induced degradation of APOPBEC3A, although sensitive to the proteasome inhibitor MG132, does not appear to require the presence of DCAF1 [45].

## 5. Conclusions and Future Directions

SAMHD1 represents a novel type of restriction factor, which is specific to cells of the myeloid lineage. Whether SAMHD1 acts as a sensor, which can then transmit an alarm signal to activate an effector mechanism, or whether it acts as an effector remains to be elucidated. SAMHD1 alone does not explain some discrepancies between cell type distribution and its ability to restrict viral infection. Therefore, it is anticipated that additional requirements for antiviral activity will soon emerge. Overcoming SAMHD1 and allowing viral integration and gene expression was observed to elicit a potent IFN response with properties that were different from those of SAMHD1-mediated restriction. Thus, it is also anticipated that further studies will eventually reveal the nature of the “cryptic sensor” present in dendritic cells.

Collectively, many studies are now highlighting an important difference in the pathogenesis between HIV-1 and that of HIV-2/SIVmac. It appears that HIV-2 and SIVmac have evolved mechanisms to overcome restriction in dendritic cells and macrophages, and can therefore efficiently infect such cell types. It is intriguing that HIV-1 can be targeted by the same restrictions and, consequently perhaps, HIV-1 infects macrophages inefficiently and does not infect dendritic cells. HIV-1 has not evolved, it seems, counteracting mechanisms that parallel the role of Vpx, but can clearly benefit from the ectopic expression of Vpx in myeloid cells.

Sound scientific discoveries raise more questions than those for which they provide answers. The identification of SAMHD1 as a myeloid cell restriction factor is undoubtedly in that category.
